# Effects of quercetin and its derivatives in *in vivo* models of neuroinflammation: A systematic review and meta-analysis

**DOI:** 10.4103/NRR.NRR-D-24-01175

**Published:** 2025-03-25

**Authors:** Michele Goulart dos Santos, Bruno Dutra Arbo, Mariana Appel Hort

**Affiliations:** 1 Programa de Pós-Graduação em Ciências Fisiológicas, Instituto de Ciências Biológicas, Universidade Federal do Rio Grande, Rio Grande, RS, Brazil; 2 Departamento de Farmacologia, Instituto de Ciências Básicas da Saúde, Universidade Federal do Rio Grande do Sul, Porto Alegre, RS, Brazil

**Keywords:** α-glycosyl isoquercitrin, alternative therapies, anti-apoptotic, antioxidant, chronic inflammation, cytokines, inflammatory mediators, neuronal damage, quercitrin

## Abstract

Neuroinflammation is an inflammatory response in the central nervous system associated with various neurological conditions. The inflammatory process is typically treated with non-steroidal and steroidal anti-inflammatory drugs, which have a range of serious adverse effects. As an alternative, naturally derived molecules such as quercetin and its derivatives show promising anti-inflammatory properties and beneficial effects on various physiological functions. Our objective was to synthesize the evidence on the anti-inflammatory effect of quercetin and its derivatives in *in vivo* models, in the face of neuroinflammatory insults induced by lipopolysaccharide, through a systematic review and meta-analysis. A search of the preclinical literature was conducted across four databases (PubMed, Web of Science, Scielo, and Google Scholar). Studies were selected based on inclusion and exclusion criteria, assessed for methodological quality using CAMARADES, and risk of bias using the SYRCLE tool, and data were extracted from the studies. The quantitative assessment of quercetin effects on the expression of pro-inflammatory cytokines and microgliosis was performed through a meta-analysis. A total of 384 potentially relevant articles were identified, of which 11 studies were included in the analysis. The methodological quality was assessed, resulting in an average score of 5.8/10, and the overall risk of bias analysis revealed a lack of methodological clarity in most studies. Furthermore, through the meta-analysis, it was observed that treatment with quercetin statistically reduces pro-inflammatory cytokines, such as tumor necrosis factor alpha, interleukin 6, interleukin 1β (*n* = 89; SMD = –2.00; 95% CI: –3.29 to –0.71), and microgliosis (*n* = 33; SMD = –2.56; 95% CI: –4.07 to –1.10). In terms of underlying mechanisms, quercetin and its derivatives exhibit antioxidant and anti-apoptotic properties, possibly through the nuclear factor erythroid 2-related factor 2 (Nrf2)/HO-1 pathways, increasing the expression of antioxidant enzymes and reducing reactive species, and modulating the caspase pathway, increasing levels of anti-apoptotic proteins and decreasing pro-apoptotic proteins. Quercetin and its derivatives exhibit highly pleiotropic actions that simultaneously contribute to preventing neuroinflammation. However, despite promising results in animal models, future directions should focus on well-designed clinical studies to assess the safety, bioavailability, and efficacy of quercetin and its derivatives in humans. Additionally, standardization of methods and dosages in studies is crucial to ensure consistency of findings and optimize their application in clinical settings.

## Introduction

Neuroinflammation is an inflammatory response of the central nervous system (CNS) triggered by various stimuli, such as infections, traumatic injuries, and neurodegenerative processes (Lukacova et al., 2021). This complex process, which involves the brain and spinal cord, includes changes to a reactive state of glial cells, such as microglia and astrocytes, and the release of cytokines, chemokines, and other pro-inflammatory molecules (Larrea et al., 2023; Ning et al., 2024). Although neuroinflammation is a natural response of the body to injuries or infections, in excessive and chronic conditions it contributes to the development or progression of several neurological and psychiatric diseases (Zhang et al., 2023).

Neuroinflammation has been implicated in Alzheimer’s disease (Zhao et al., 2019), Parkinson’s disease (PD) (Araújo et al., 2022), amyotrophic lateral sclerosis (He et al., 2024), autism (Hughes et al., 2023), depression (Troubat et al., 2021), brain cancer (Vandenbark et al., 2021), and other diseases. These pathological conditions represent a significant public health challenge, as they have high prevalence, a substantial impact on quality of life, and great complexity in treatment (Feigin et al., 2020). Many of these diseases, such as Alzheimer’s disease, PD, and amyotrophic lateral sclerosis, still have no cure, and the available treatments are generally palliative, focusing on symptom management and slowing disease progression (Poewe et al., 2017; Masrori and Van Damme, 2020; Yiannopoulou and Papageorgiou, 2020).

Lipopolysaccharide (LPS) is present in the cell wall of gram-negative bacteria, being widely used in experimental models of neuroinflammation (Skrzypczak-Wiercioch and Sałat, 2022). In the CNS, it is recognized by the Toll-like receptor 4 (TLR4/CD14) complex expressed in microglial cells, triggering a series of signaling pathways and leading to the increase of pro-inflammatory cytokines, such as interleukin 1 (IL-1) and 6 (IL-6), tumor necrosis factor alpha (TNF-α) and other inflammatory mediators such as cyclooxygenase (COX-2), prostaglandin E2, and nitric oxide (NO), among others (Mehramiz et al., 2023). Furthermore, numerous studies have reported that the activation of glial cells not only releases inflammatory mediators but also produces reactive oxygen species (ROS), which also leads to neurodegeneration mediated by neuroinflammation (Teleanu et al., 2022). LPS-induced inflammation in *in vivo* models has been widely used in the research of new anti-inflammatory drugs and promising molecules for controlling inflammation (Markov et al., 2020).

Conventional treatments for inflammation include the use of non-steroidal anti-inflammatory drugs (NSAIDs) and steroids (Patil et al., 2019). However, the chronic use of NSAIDs may result in gastrointestinal problems, including the development of gastric and duodenal ulcers, as well as severe hemorrhages that can be fatal (Bindu et al., 2020). Additionally, the use of high doses of anti-inflammatory drugs is associated with significant bone loss because these drugs inhibit osteoblast activity and increase osteoclast activity. This imbalance leads to osteoporosis and raises the risk of fractures due to increased bone fragility (Chotiyarnwong and McCloskey, 2020). Glucocorticoids also exert catabolic effects on muscles, promoting the degradation of muscle proteins, such as myosin, and inhibiting protein synthesis, and their prolonged use could be associated with progressive loss of muscle mass and strength (Haran et al., 2018; Lee et al., 2022). Other associated harms include hypertension and obesity, as glucocorticoids can increase insulin resistance, cause dyslipidemia, and promote fat accumulation (Lengton et al., 2022), and behavioral and cognitive changes, affecting key areas such as the hippocampus and the prefrontal cortex, leading to memory deficits (Koning et al., 2024).

**Figure 1:**
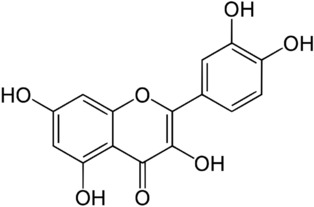
Structural representation of quercetin. Source: https://pubchem.ncbi.nlm.nih.gov/.

The study of naturally derived molecules emerges as a promising alternative in the search for new drugs with anti-inflammatory properties and fewer adverse effects. A naturally occurring molecule that has been studied for its anti-inflammatory properties is quercetin (QUE) 2-(3,4-dihydroxyphenyl)-3,5,7-trihydroxychromen-4-one (**Figure 1**). Belonging to the flavonol family, it is a natural compound found in various food sources, such as cherries, apples, red wine, capers, and red onions (van der Meulen et al., 2022).

QUE has numerous beneficial properties and plays a role in several physiological functions. Its anti-inflammatory effects are associated with decreasing the production of pro-inflammatory cytokines such as TNF-α, IL-1β, and IL-6 (Hannan et al., 2023). Furthermore, QUE inhibits the production of NO (Kukula and Günaydin, 2023), inactivates the nuclear factor kappa (NF-κB) signaling pathway, a key mediator in neuroinflammation (Das et al., 2024), modulates the mitogen-activated protein kinase (MAPK) pathway (Kedhari Sundaram et al., 2019), and reduces astrogliosis and microgliosis (Tsai et al., 2021). In addition to its anti-inflammatory effects, QUE exhibits potent antioxidant properties, protecting neurons from oxidative stress-induced damage (Xu et al., 2019; de Oliveira Vian et al., 2024). It enhances the expression of key antioxidant enzymes, such as superoxide dismutase and catalase (CAT), which play crucial roles in neutralizing ROS and mitigating oxidative stress linked to inflammation. All these mechanisms make QUE a promising candidate for the treatment of damage associated with inflammation and oxidative stress.

Despite the numerous properties of QUE, the molecule also presents several factors that limit its clinical use, such as low water solubility, consequently leading to low bioavailability in the body, rapid clearance, metabolism, and enzymatic degradation (Riva et al., 2019). Therefore, various modified dosage forms of the molecule, primarily nanoparticles, microemulsions, and other delivery vehicles (Niazvand et al., 2019), have been developed to minimize these limitations. Additionally, QUE derivatives, such as glycosides and sulfate derivatives, are being extensively studied due to their improved bioavailability and potential therapeutic applications (Aljadaan et al., 2020; Alizadeh et al., 2023). Research has shown that these QUE derivatives retain many of the biological activities of the original compound, such as the ability to modulate important inflammatory and antioxidant pathways (Lesjak et al., 2018; Rogovskii et al., 2021).

In this context, systematic reviews and meta-analyses of preclinical trials have become valuable research tools, as they compile all primary research that meets predefined qualification criteria, allowing specific research questions to be addressed with reduced bias. Here, we conducted a systematic review and meta-analysis to provide compelling evidence on the anti-neuroinflammatory effects of QUE and its derivatives in *in vivo* models, highlighting their relevance as potential therapeutic targets in response to inflammatory insults and the importance of guiding future clinical trials in this area.

## Methods

The question that guided this study was “What are the anti-inflammatory effects of QUE and its derivatives in *in vivo* models of LPS-induced neuroinflammation?” based on the abbreviation PICOS (da Costa Santos et al., 2007), which is composed of: population (P): animals subjected to the neuroinflammation model with LPS; intervention (I): QUE and its derivatives; comparator (C): the control group (animals treated with LPS and that did not receive any other intervention); outcomes (O): anti-inflammatory effects presented by QUE and its derivatives; type of study (S): *in vivo* (non-human animals). This work was registered in International Prospective Register of Systematic Reviews (PROSPERO) under number CRD42023416284, and conducted in accordance with the Preferred Reporting Items for Systematic Reviews and Meta-Analyses (PRISMA) guidelines (Page et al., 2021).

### Literature search

Two researchers independently searched in early May 2024 across the following databases: PubMed, SciELO, Web of Science, and Google Scholar (for gray literature). These databases were queried using keywords and their combinations, such as all MeSH terms and two more “Entry Terms” that were the two main synonyms frequently referenced in PubMed, they are: ““Quercetin”[Mesh] OR 3,3’,4’,5,7-Pentahydroxyflavone OR Dikvertin” AND (“Neuroinflammatory Diseases” [Mesh] OR Disease, Neuroinflammatory OR Neuroinflammatory Disease OR Neuroinflammatory Disorders OR Disorder, Neuroinflammatory OR Neuroinflammation OR Neuroinflammations). The records retrieved from each database and website were exported to the Rayyan platform (https://www.rayyan.ai/) for semi-automatic duplicate removal and eligibility analysis. After the duplicates were removed, two reviewers read the titles and abstracts, and in cases of disagreement, a third reviewer was consulted. After selecting the studies that met the pre-established criteria, a second evaluation was conducted, involving a full-text analysis, to finalize the list of articles that met all the inclusion criteria.

### Inclusion criteria

Studies that evaluated the anti-neuroinflammatory effects of QUE and its derivatives, at different doses, forms of administration, frequency of administration, and time in *in vivo* experimental models induced by LPS, using several species of animals, were included in the review. The language was limited to English, Spanish, or Portuguese, with no restrictions on the year of publication.

### Exclusion criteria

*In silico*, *in vitro*, and human studies, as well as *in vivo* studies induced by models other than LPS, were excluded. For *in vivo* models, interventions with other chemical substances or in combination with QUE and derivatives, studies that did not have a control group (animals untreated or treated with vehicle), and transgenerational studies were also excluded. Other types of documents, such as theses, reviews, and conference abstracts, were excluded.

### Data extraction

Study data were extracted independently by two researchers and are presented in **Additional Table 1**, which contains the following information: (1) first author (year); (2) animal data; (3) weight, and age; (4) Quercetin/ Derivatives (dose/route of administration); (5) quercetin/derivatives treatment (type/exposure time/frequency); (6) LPS (dose/time/route of administration); (7) methods/evaluated parameters; (8) anti-neuroinflammatory activities (primary outcomes); (9) secondary outcomes, that refer to outcomes other than inflammation directly.

**Table 1:** Quality assessment of included studies

CAMARADES checklist (adapted)	Adeoluwa et al., 2023	Hussein et al., 2024	Kang et al., 2020	Khan et al., 2018	Lee et al., 2020a	Su et al., 2024	Singh et al., 2022	Tang et al., 2023	Han et al., 2021	Sun et al., 2021b	Zou et al., 2024
A	√	√	√	√	√	√	√	√	√	√	√
B	√	√	√	√	√		√	√	√	√	√
C											
D											
E					√			√	√	√	
F	√			√	√	√	√	√	√		√
G	√	√	√	√	√	√	√	√	√	√	√
H											
I	√	√	√	√	√	√	√	√	√	√	√
J		√	√	√	√	√	√	√	√		√
Score	5	5	5	6	7	5	6	7	7	5	6

A: Publication in peer-reviewed journal; B: statement of control of temperature; C: randomization of treatment or control; D: allocation concealment; E: blinded assessment of outcome; F: avoidance of anesthetics with marked intrinsic properties; G: use of animals with appropriate neuroinflammation model; H: sample size calculation; I: statement of compliance with regulatory requirements; J: statement regarding possible conflict of interest.

### Quality assessment and risk of bias

To evaluate the potential for bias in the included studies, the SYRCLE risk of bias tool (Hooijmans et al., 2014; McGuinness and Higgins, 2021) was applied. This tool comprises 10 criteria addressing selection bias, performance bias, detection bias, attrition bias, and reporting bias. The methodological quality of the studies was evaluated using the Collaborative Approach for Meta-Analysis and Review of Animal Data from Experimental Studies (CAMARADES), with items adapted for this work (Zeng et al., 2015). The adapted CAMARADES criteria encompass the following guidelines for *in vivo* studies: (A) publication in a peer-reviewed journal; (B) declaration of temperature control; (C) randomization of treatment or control, with a description of the method; (D) allocation concealment; (E) blinded assessment of at least one outcome; (F) avoidance of anesthetics with marked intrinsic properties; (G) use of appropriate neuroinflammation model animals; (H) sample size calculation; (I) declaration of compliance with regulatory requirements; (J) declaration of potential conflict of interest. One point was awarded for each criterion met, and zero if the information was missing, insufficient, or unclear, resulting in a final score ranging from 0 (lowest) to 10 (highest). For the quality assessment score, the range of scores and the overall mean across studies were reported. The grading of the assessment criteria was conducted by two independent authors, and any disagreements were resolved through discussion.

### Quantitative synthesis and statistical analyses

Quantitative data were analyzed using R software version 4.4.1 for Windows, with the meta package version 7.0-0. For the continuous variables of interest, the mean score and standard deviation and/or standard error of the mean, for both the treatment and model groups (animals treated only with LPS), were extracted using WebPlotDigitizer from a publicly available plot, as the results were presented graphically and not as numerical data. The effect size of QUE administration on the expression of pro-inflammatory cytokines (IL-6, IL-1β, and TNF-α), and the effect of microgliosis through the Iba-1 marker, for each outcome in each study was measured using the standardized mean difference (*SMD*), by Hedge’s G method (Hedges and Pigott, 2001), and the meta-analyses were conducted using a random-effects model by DerSimonian and Laird (1986). The results are expressed as effect size ± 95% confidence interval (CI), with analyses showing *P* < 0.05 considered statistically significant. In cases where the same control group served as a comparator for multiple experimental groups within a single study, the control group sample size was divided by the number of comparisons included in the meta-analysis. Heterogeneity was evaluated using the *I*² statistic, where an *I*² value near 0% suggests no heterogeneity between studies, around 25% indicates low heterogeneity, about 50% signifies moderate heterogeneity, and close to 75% reflects high heterogeneity among studies (Zhao, 2013).

## Results

### Research screening

**Figure 2:**
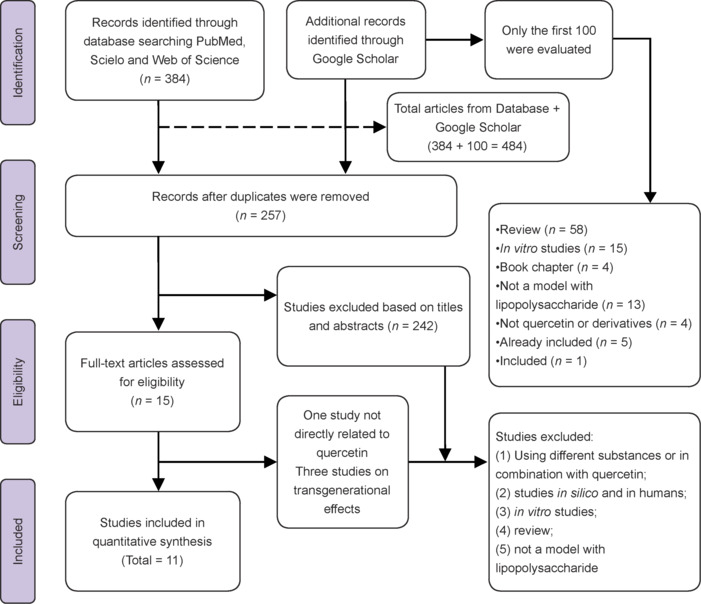
Flow chart of paper identification and inclusion process.

Following the comprehensive search strategy, a total of 384 records were found across the PubMed, Scielo, and Web of Science databases, along with an analysis of the first 100 records from Google Scholar, which are ordered by relevance by the algorithm. Upon importing the references into Rayyan, 127 duplicates were semi-automatically removed, and the eligibility of 257 articles was collaboratively analyzed. After screening titles and abstracts, 242 studies were excluded because they met at least one of the exclusion criteria: (1) Studies involving molecules other than QUE or its derivatives, or in combination; (2) *in silico* or human studies; (3) *in vitro* studies; (4) literature reviews or book chapters; (5) studies that did not involve LPS-induced neuroinflammation models. The remaining 15 records were downloaded for further full-text manuscript analysis. Based on the eligibility criteria, four records were removed: one due to the treatment group involving acylated isoquercetin derivatives, which were neither QUE nor direct derivatives of the molecule, and three other studies focused on transgenerational effects, where the treatment was administered to the mothers, and the analyses were performed on the offspring, specifically focusing on neurodevelopment. Finally, 11 studies met the inclusion criteria for the review (**Figure 2**).

### Characteristics of included studies

**Figure 3:**
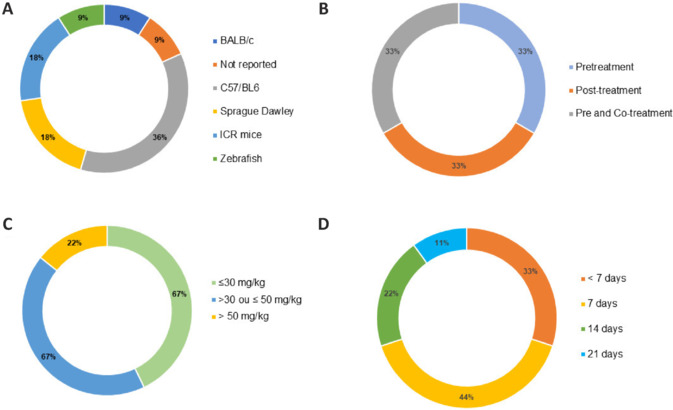
Characteristics of eligible studies. (A) Species and model strain distribution; (B) type of quercetin (QUE) treatment; (C) doses used in QUE studies; and (D) duration of QUE treatment intervention.

**Additional Table 1** summarizes the main characteristics and results of the 11 *in vivo* studies. Most experiments were conducted in different rodent models (**Figure 3A**), including 64% in mice (C57Bl/6, 4/11; BALB/c, 1/11; ICR, 2/11), 27% in rats (Sprague-Dawley, 2/11 and 1/11 did not specify the rat strain.), 9% in zebrafish (*Danio rerio*, 1/11). Regarding sex (**Figure 3B**), male animals comprised the predominant population (82%, 9/11), while 18% of studies used both male and female animals (2/11). The daily dose of QUE ranged from 1 mg/kg to 100 mg/kg of body weight (under ≤ 30 mg/kg in 6 studies, between 30 and ≤ 50 mg/kg in 2 studies, higher than > 50 mg/kg in 2 studies) (**Figure 3C**), administered primarily via intraperitoneal injection (66%, 6/9), followed by oral administration (44%, 4/9). The studies were equally divided between QUE pretreatment (33%, 3/9), posttreatment (33%, 3/9), and both pre- and cotreatment (33%, 3/9). The duration of QUE intervention ranged from 3 to 21 days, with most studies using a 7-day treatment regimen (44%, 4/9), followed by less than a week (< 7 days, 33%, 3/9), 14 days (22%, 2/9), and 21 days (11%, 1/9) (**Figure 3D**). Only one study (11%) compared the effect of free QUE with poly(lactic-co-glycolic acid) nanoparticles loaded with QUE against LPS-induced neurotoxicity in mice. Of the studies that used QUE derivatives, one utilized α-glycosyl isoquercitrin (AGIQ) at 0.5% (w/w) in the animal’s diet, which was treated for 5 weeks, while the other used quercitrin (QC) at 10 mg/kg, administered intraperitoneally for up to 8 weeks.

Among the primary outcomes analyzed, 8 studies (73%) evaluated the effects of QUE and/or its derivatives on the expression of pro-inflammatory cytokines. Additionally, 5 studies (45%) evaluated inducible nitric oxide synthase (iNOS) levels, 3 studies (27%) evaluated COX-2 levels, mainly through enzyme-linked immunosorbent assay, western blotting, or polymerase chain reaction techniques. Three studies (27%) examined the effect of these molecules on astrocyte activation via glial fibrillary acidic protein expression, while microgliosis was assessed in five studies (45%) using the Iba-1 marker, primarily through immunohistochemistry. Furthermore, four studies (36%) evaluated NF-κB expression, two studies (18%) examined NLRP3 expression, and one study (9%) assessed nitrite levels and TLR4 receptor expression, using the aforementioned techniques. Of the secondary parameters evaluated, the majority of studies (73%, 8/11) conducted behavioral tests such as the forced swimming test, open field test, tail suspension test, Morris Water Maze task, Y-maze task, and rotarod test. Oxidative stress parameters were also assessed in 64% of the studies (3/11), including levels of lipid peroxidation, reduced glutathione (GSH), malondialdehyde, CAT, and Nrf2 expression, among others. Apoptotic markers were evaluated in 18% of the studies (2/11), focusing on the expression of caspase-1, Bcl-2, Bax, and other related factors. Additionally, all studies assessed at least one other secondary parameter, such as acetylcholinesterase activity, gamma-aminobutyric acid levels (GABA), tyrosine hydroxylase, and others.

### Methodological quality and risk of bias of studies

The quality of the 11 included studies was assessed using the adapted 10-item CAMARADES checklist (**Table 1**). The average quality score was 5.8, with a range of scores from 5/10 to 7/10. Among these, most studies scored 5 points (45%, 5/11), while the studies by Khan et al. (2018), Singh et al. (2022), and Zou et al. (2024) received 6 points (27%), and the studies by Han et al. (2021), Lee et al. (2020a), and Tang et al. (2023) received 7 points (27%). Overall, all included records were peer-reviewed, stated the temperature conditions, and used appropriate animal models for neuroinflammation and avoidance of anesthetics with marked intrinsic properties. Seven studies reported randomization of animals, but did not specify how, as did studies that did not perform this process. None of the records reported on allocation concealment, nor was there mention of a formal sample size calculation. Four studies mentioned blinding in at least one of the analyses performed, so this criterion was scored (Lee et al., 2020a; Han et al., 2021; Sun et al., 2021b; Tang et al., 2023). All studies declared compliance with regulatory requirements, and only two studies (18%) did not report details about conflicts of interest. It is important to highlight that there was a lack of methodological information in the selected studies, particularly regarding the randomization method, unclear details about blinding (if any) in all analyses, and allocation concealment.

**Figure 4:**
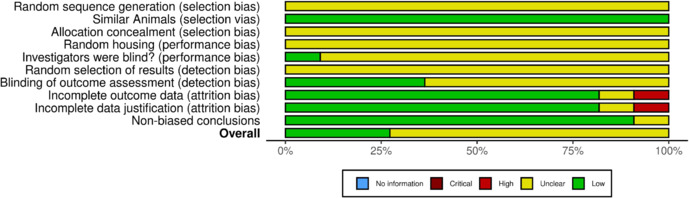
Risk of bias from included studies.

The risk of bias assessment is shown in **Figure 4**; it was conducted based on the criteria established by the SYRCLE tool. A judgment of “Low” indicates a low risk of bias, while a judgment of “High” indicates a high risk of bias. If the report lacked sufficient details to assess any of the parameters, the judgment was “Unclear.” Through this analysis, we observed a lack of detailed information about the experimental design in most studies, particularly concerning the method used for random sequence generation, allocation concealment, random housing of animals, random selection of outcomes (detection bias), concealment of outcome assessment, and blinding of outcome assessment. Some studies were also unclear about investigator blinding during the experiment (performance bias). Therefore, a “Low” judgment was applied for this criterion when the authors explicitly stated that both caregivers and researchers were blinded to the interventions assigned to the animals throughout all experiments, ensuring they had no knowledge of which animals received specific treatments. An “Unclear” judgment was applied when blinding was only mentioned for one outcome assessment and/or test. As a result, the judgment ‘Unclear’ was the most prevalent in the description of the results (75%).

### Meta-analysis results

**Figure 5:**
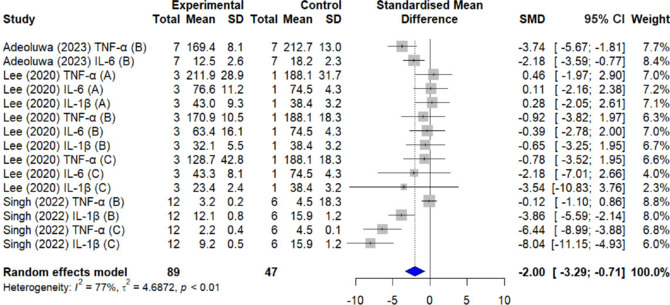
Forest plot (effect size and 95% CI) summarizing the effect of quercetin on proinflammatory cytokine levels. IL: Interleukin; TNF: tumor necrosis factor.

The random effects model was used for all meta-analyses. Statistical heterogeneity among the three included studies showed an *I*² index of 77%, and the combined results of the meta-analysis demonstrated that QUE administration significantly reduced pro-inflammatory cytokine levels (*n* = 89; SMD = –2.00; 95% CI: –3.29 to –0.71; *τ*² = 4.6872; *P* < 0.01) compared with the control (untreated) group of LPS-induced animals (**Figure 5**).

**Figure 6:**
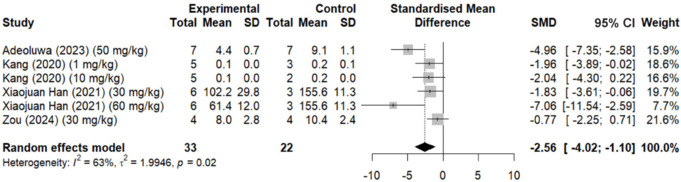
Forest plot (effect size and 95% CI) summarizing the effect of quercetin on the microgliosis marker ionized calcium binding adaptor molecule 1.

Regarding the effect of QUE treatment on microgliosis, measured through the Iba-1 marker, four studies were selected for the meta-analysis, with SMDs ranging from –0.77 to –7.06 (*n* = 33; SMD: –2.56), suggesting that all studies indicate a reduction in the Iba-1 marker in the experimental group compared with the control group. The 95% CI for most studies does not include the value 0 (–4.07 to –1.10), indicating that these reductions are statistically significant. The heterogeneity among the studies is moderate, with an *I*² of 63% (*τ*² = 1.9946, *P* = 0.02; **Figure 6**).

## Discussion

The inflammatory process is activated to combat and control injuries, infections, or other stimuli, promoting the repair of damaged tissues and the elimination of pathogenic agents. However, when this response becomes continuous, it can create a chronic inflammatory environment, leading to progressive tissue damage (Solleiro-Villavicencio and Rivas-Arancibia, 2018). Neuroinflammation refers to the inflammatory response of the CNS to factors that disrupt its homeostasis, involving the action of glial cells such as microglia, oligodendrocytes, astrocytes, dendritic cells, and peripheral leukocytes (Kölliker-Frers et al., 2021). This response is observed in a range of neurological disorders, such as metabolic, toxic, ischemic, infectious, neurodegenerative, and neoplastic conditions (French et al., 2019; Krämer et al., 2019; Asslih et al., 2021; Tansey et al., 2022). Given the crucial role of neuroinflammation in the onset and progression of these diseases, it is essential to understand and control this response to prevent or slow down the progression of CNS disorders.

Among the most important and widely used animal models for studying neuroinflammation, and neurodegeneration are those employing LPS (Skrzypczak-Wiercioch and Sałat, 2022). This model has become a robust tool for investigating the mechanisms underlying neurodegeneration and neuroinflammatory diseases, since LPS administration causes an inflammatory response mediated mainly by TLR4 receptors, microglial reactivation and neuroinflammation, which results in neuronal degeneration, synaptic loss, and finally neuronal cell death (Yang et al., 2020).

In this systematic review, the effects of QUE and its derivatives were examined in 11 pre-clinical *in vivo* studies of LPS-induced neuroinflammation. The included studies span from 2018 to 2024, indicating that the investigation of the anti-inflammatory effects of QUE and its derivatives in LPS models is relatively recent. The main findings of this systematic review suggest that QUE and its derivatives AGIQ and QC may exert significant anti-neuroinflammatory effects, potentially mediating their actions through modulation of inflammatory pathways, prevention of apoptosis, and reduction of oxidative stress.

### Anti-inflammatory properties of quercetin and derivatives

Most studies used doses of QUE ranging from 10 to 50 mg/kg, with the majority implementing a pre-treatment period of at least 7 days, with prolonged exposures extending up to at least 21 days. QUE is known to be a lipophilic substance, so prolonged pre-treatment helps to ensure that the molecule reaches therapeutic concentrations in the brain. Furthermore, continuous treatment can progressively inhibit inflammatory cascades, allowing the anti-inflammatory effects of QUE to be maximized (Mehany et al., 2022). Although QUE is a lipophilic substance capable of crossing the blood–brain barrier and prolonged treatment can help QUE reach effective therapeutic concentrations, some studies have explored delivery systems such as nanoparticle encapsulation, which improve the molecule’s bioavailability and its distribution to brain tissues (Pinheiro et al., 2021; Tomou et al., 2023). These systems help QUE accumulate in the brain more efficiently, making continuous treatment more effective in inhibiting inflammatory cascades over time.

Hussein et al. (2024) examined the neuroprotective effect of poly(lactic-co-glycolic acid) nanoparticles loaded with QUE against LPS-induced neurotoxicity in mice and found that the neuroprotective effect of nanoparticles loaded with QUE was significantly greater than free QUE in several parameters, such as its anti-inflammatory action, as it reduced iNOS levels, an enzyme responsible for producing large amounts of NO (Iova et al., 2023). This overexpression of iNOS has been associated with neurodegenerative diseases such as Alzheimer’s disease, multiple sclerosis, and PD (Barua et al., 2019), where chronic neuroinflammation is an important contributor to the progression of these pathologies

In neuroinflammation, iNOS expression is induced by pro-inflammatory cytokines, which are proteins released mainly by immune cells, such as monocytes, macrophages, and lymphocytes, as well as microglia and astrocytes in the CNS (Kim et al., 2016). Among the nine studies that conducted treatment with QUE, six evaluated its effect on the expression of pro-inflammatory cytokines (IL-17, IL-6, IL-1β, and TNF-α). IL-17 stimulates the production of other pro-inflammatory cytokines, such as IL-6, IL-1β, and TNF-α, and induces the production of chemokines that recruit neutrophils to the site of infection, amplifying the inflammatory response (Milovanovic et al., 2020). IL-6 is a soluble cytokine with pleiotropic effects on inflammation, produced by various cell types, and is crucial for the immune and inflammatory response (Grebenciucova and VanHaerents, 2023). It acts as a mediator, signaling the occurrence of an emergent event, such as an infectious injury or tissue damage, and sends an alert signal throughout the body, promoting direct or indirect inflammation (Kummer et al., 2021). 

The IL-1β is a potent pro-inflammatory cytokine essential for host defense responses to infections and injuries (Pyrillou et al., 2020). It is the most extensively studied and well-characterized member among the 11 proteins in the IL-1 family (Pyrillou et al., 2020). After being activated by the inflammasome, an intracellular protein complex, this cytokine is secreted and triggers an inflammatory cascade by stimulating the production of other cytokines, such as IL-6 and TNF-α, in addition to promoting the recruitment of immune cells to the site of infection or injury. Furthermore, it induces fever by acting on the hypothalamus, increasing body temperature during systemic inflammation (Mota and Madden, 2022). Another key mediator in inflammation in many chronic systemic inflammatory and degenerative conditions is TNF-α, which, when binding to its receptor, promotes the induction of NF-κB signaling or cytotoxicity through apoptosis or necroptosis (Jayaraman et al., 2021).

The combined results of the meta-analysis, restricted to studies that used the same technique, in this case, the enzyme-linked immunosorbent assay, demonstrated that QUE reduced the production of these cytokines, thereby minimizing damage associated with chronic inflammation and tissue injury. These cytokines can cross the blood–brain barrier and are also produced in the CNS by glial cells such as microglia, which regulate inflammatory processes in the brain. Moreover, the reduction of pro-inflammatory cytokines in the CNS not only mitigates oxidative stress but also helps prevent the progressive neuronal damage commonly observed in neurodegenerative diseases where chronic neuroinflammation is a key pathological feature (Prasad, 2017). This makes QUE a promising molecule for the treatment of conditions associated with neuroinflammation.

This reduction can be explained by several cellular and molecular mechanisms, such as the inhibition of the NF-κB signaling pathway, preventing its translocation to the nucleus and the subsequent transcription of inflammatory genes (Gao et al., 2020), modulation of the MAPK pathway through the activation of protein kinases such as ERK, JNK, and p38 (Sahu and Rawal, 2024), inhibition of NLRP3 inflammasome activation, decreasing cytokine production, and activation of the Nrf2 pathway, which regulates the expression of antioxidant enzymes against oxidative stress and consequently reduces ROS (Ngo and Duennwald, 2022). These mechanisms have also been investigated in some of the included studies, such as the reduction of NF-κB (Khan et al., 2018; Lee et al., 2020a; Adeoluwa et al., 2023), reduction of the NLRP3 inflammasome (Han et al., 2021; Adeoluwa et al., 2023), ERK (Zou et al., 2024), and TLR4 (Khan et al., 2018), thus blocking excessive activation of the inflammatory response.

During neuroinflammation, glial cells such as astrocytes and microglia play essential roles in maintaining brain homeostasis and responding to injuries and diseases. However, the effect of these cells depends on their activation state, which can be either pro-inflammatory or neuroprotective (Zhang et al., 2023). As mentioned previously pro-inflammatory cytokines can activate previously resting glial cells into a reactive state, leading to the release of additional inflammatory factors such as interleukins, NO, and proteases, which cause damage to the CNS (Kölliker-Frers et al., 2021).

The identification of reactive astrocytes and microglia can be done through the expression of specific markers that indicate their activation and changes in their functional state. For example, glial fibrillary acidic protein is one of the most commonly used markers for reactive astrocytes, while Iba-1 is used for microglia, with the expression of these markers increasing upon activation (Kwon and Koh, 2020). In this review, it was observed that QUE significantly reduced the expression of both glial fibrillary acidic protein (Khan et al., 2018; Kang et al., 2020) and Iba-1 (Khan et al., 2018; Kang et al., 2020; Han et al., 2021; Zou et al., 2024). Furthermore, the reduction in microglial activation was confirmed in the meta-analysis of the four studies, and the combined results indicated a reduction in the Iba-1 marker in groups treated with different doses of QUE (1–60 mg/kg) compared with the LPS group. As previously mentioned, these reactive cells can activate inflammatory enzymes such as iNOS and COX-2. Therefore, in addition to reducing microgliosis and astrogliosis, QUE has also been observed to reduce the expression of iNOS and COX-2 in studies (Khan et al., 2018; Lee et al., 2020a; Hussein et al., 2024).

Among the two studies with QUE derivatives, one used the AGIQ derivative at a concentration of 0.5% in the animals’ diet (Tang et al., 2023). Significant reductions in TNF-α and COX-2 expression were observed; however, for other analyses, such as gene expressions of GABAergic interneuron markers, neurotrophic factors, synaptic plasticity, glutamate receptor, and transporter genes, and cholinergic receptor genes—no effects of this molecule were noted compared with the LPS-treated group.

The other glycosylated flavonoid investigated was QC, which has antioxidant, anti-inflammatory, and neuroprotective properties similar to those of QUE, but with differences in bioavailability and metabolism (Park et al., 2018). Sun et al. (2021b) administered a single dose of QC (10 mg/kg) and achieved promising results, such as reductions in the expression of IL-1β, IL-10, TNF-α, and hippocampal signaling pathways of PI3K/AKT/NF-κB and MAPK. This indicates that this derivative may reduce inflammation and modulate signaling pathways related to stress and inflammation in the hippocampus, demonstrating its therapeutic potential similar to QUE.

### Antioxidant properties of quercetin and derivatives

Oxidative stress plays a key role in regulating the inflammatory response, with the presence of ROS leading to neuronal damage and the activation of microglia and astrocytes, in addition to oxidizing proteins and lipids, and damaging DNA (Solleiro-Villavicencio and Rivas-Arancibia, 2018). Several preclinical studies have already demonstrated the antioxidant effects of QUE (Priyanga et al., 2017; Lesjak et al., 2018; Xu et al., 2019). The studies included in this review showed that QUE reduced lipid peroxidation by decreasing levels of malondialdehyde (Singh et al., 2022; Hussein et al., 2024), which is known as a reliable biomarker of oxidative stress with highly reactive instability that can attack macromolecules.

Treatment with QUE also promoted an increase in antioxidant enzymes such as CAT, HO-1 (Hussein et al., 2024), and GSH (Singh et al., 2022; Hussein et al., 2024), which are essential in the defense against ROS by eliminating free radicals and reducing H₂O₂. The positive regulation of these enzymes helps mitigate the harmful effects of ROS. The study that utilized nano-encapsulated QUE, nanoparticles loaded with QUE increased the concentrations of GSH, CAT, and enhanced the expression of the Nrf2 and HO-1 genes (Hussein et al., 2024). These effects are crucial for reducing neuronal damage and enhancing the expression of key antioxidant enzymes such as GSH, superoxide dismutase, and CAT, which play a central role in neutralizing free radicals. This mechanism offers protection to DNA, proteins, and other biomolecules from the damaging effects of ROS (Lee et al., 2020b).

The main mechanisms by which QUE exerts its antioxidant effects can be explained by its chemical structure, rich in hydroxyl groups (-OH), which allows it to neutralize free radicals (Veiko et al., 2021). Moreover, QUE can inhibit pro-oxidant enzymes and activate the Nrf2 pathway, an essential antioxidant mediator that plays a crucial role in protection against oxidative stress (Sadi et al., 2014; Uddin et al., 2021). QUE inhibits the interaction of Nrf2 with Keap1, a repressive protein that normally degrades Nrf2, by interacting with the Keap1-Nrf2 complex, prevents this degradation, leading to the stabilization and nuclear translocation of Nrf2 (Ebrahimi et al., 2023). Once in the nucleus, this factor positively regulates several antioxidant response elements, suggesting that QUE may significantly inhibit oxidative stress and reduce the neurotoxicity of ROS.

Other studies suggest that QUE’s antioxidant effects may also be attributed to its ability to activate the SIRT1 (Sirtuin 1) pathway, an enzyme from the sirtuin family involved in the regulation of cellular processes such as aging, inflammation, metabolism, and oxidative stress response (Zhang et al., 2020; Cui et al., 2022; Mehramiz et al., 2023). SIRT1 activation can increase the expression of genes encoding antioxidant enzymes, while also promoting cell survival through mitochondrial biogenesis, improving mitochondrial efficiency, and reducing oxidative stress in the long term (Mehramiz et al., 2023).

Among the two studies involving QUE derivatives, biochemical parameters of oxidative stress were evaluated only in the study by Tang et al. (2023), which found that the AGIQ derivative was not able to reduce malondialdehyde levels induced by LPS. The addition of a glycosyl group to the QUE core can alter its absorption, distribution, and metabolism in the body. While glycosylation improves water solubility and facilitates gastrointestinal absorption, it may reduce its ability to penetrate and achieve effective concentrations in the CNS. Additionally, the presence of these glycosyl groups can affect the reactivity and stability of the molecule and alter its ability to interact with ROS (Sun et al., 2021a).

### Other mechanisms related to neuroprotection of quercetin and its derivatives

Neuroinflammation is associated with various pathological conditions that negatively affect behavior and cognition. The studies included in this review assessed the effects of QUE and its derivatives through behavioral tests to identify their impact on cognitive and behavioral deficits in animals subjected to neuroinflammatory insult. These tests allow the analysis of cognitive deficits, stress levels, and emotional behavior in rodents, which often reflect neuropsychiatric and neurodegenerative conditions such as depression and dementia, associated with neuroinflammation.

The forced swimming test was used in some studies (Han et al., 2021; Sun et al., 2021b; Adeoluwa et al., 2023) and is founded on the premise that, when placed in a water-filled container, an animal will initially attempt to escape but will ultimately display immobility, interpreted as an indicator of behavioral despair (Yankelevitch-Yahav et al., 2015). This test has been widely used because it involves exposing animals to stress, which has been shown to play a role in the tendency toward severe depression. Animals with high levels of neuroinflammation may show increased immobility time, suggesting a depressive-like response. It was observed that QUE and its derivative QC significantly reduced the immobility time of the animals compared with the LPS group. Other tests were also evaluated, such as the tail suspension test, which is similar to the forced swimming test. In tail suspension test, animals are suspended by their tails, which is a stressful situation for the animal. The lack of escape-related behavior is considered immobility, and this is how the time is measured (Can et al., 2012). QUE treatment significantly reduced this time compared with the LPS group, and treatment with QC also showed similar results (Han et al., 2021; Sun et al., 2021b; Su et al., 2024).

Some studies also performed the open field test, which evaluates locomotor activity and anxiety behavior in a new and open environment (Lee et al., 2020a; Sun et al., 2021b; Adeoluwa et al., 2023; Tang et al., 2023; Su et al., 2024). Rodents with neuroinflammation often show reduced exploration and a greater tendency to stay near the edges of the field, a typical anxiety-related behavior. However, treatment with QUE reduced this behavior (Lee et al., 2020a; Adeoluwa et al., 2023; Su et al., 2024), while treatment with the derivatives AGIQ and QC did not produce any change (Sun et al., 2021b; Tang et al., 2023). An adaptation of this test was conducted in zebrafish, called the Time in novel tank diving test, also to assess anxiety levels and exploratory behavior. It was observed that QUE increased the time spent in the upper zone of the tank, suggesting less anxious behavior, as anxious fish tend to remain in the lower part of the tank for longer (Singh et al., 2022).

Khan et al. (2018) were the only ones to evaluate the effects of LPS in behavioral tests that assess memory. The first was the Morris Water Maze task, widely used to evaluate spatial memory and learning in rodents. The animal must learn to locate a submerged platform in a circular pool using spatial cues. In the study, LPS-injected animals took more time to locate the hidden platform compared with control mice. However, QUE treatment reversed the LPS-induced effect and significantly enhanced memory. The second memory test was the Y-maze task, in which rodents must alternate between the arms of the maze. The time spent in the “lower zone” can reflect memory deficits and hesitation behavior, often exacerbated by brain inflammation. Y-maze results showed that LPS impaired short-term spatial memory compared with the control group. QUE treatment significantly increased the percentage of spontaneous alternations, indicating that it improved working spatial memory in LPS-exposed mice.

In addition to behavioral parameters, other outcomes were also investigated, such as anti-apoptotic effects. QUE exerts this effect through several mechanisms, among which is its ability to modulate the caspase pathway, which includes key enzymes involved in the execution of the apoptotic process (Yu et al., 2024). Here, it was observed that QUE inhibited the activation of caspases-3 and -1, which are central enzymes in the apoptotic pathway (Khan et al., 2018; Han et al., 2021). Caspase-3 is considered an effector caspase, being activated in the final stages of the apoptotic pathway, through either the intrinsic (mitochondrial) or extrinsic (death receptor-mediated) pathway (Yu et al., 2024). Caspase-1 plays a crucial role in the inflammatory response and programmed cell death, with its main function being the activation of pro-inflammatory cytokines such as IL-1β and IL-18, promoting the inflammatory response (Yu et al., 2024). In addition to modulating the caspase pathway, QUE regulates Bcl-2 family proteins, which control mitochondrial membrane permeability. Increases in levels of anti-apoptotic proteins, like Bcl-2, and reductions in pro-apoptotic proteins, like Bax, were also observed, as well as reduced cytochrome C release, thereby preventing cell death (Khan et al., 2018).

### General characteristics and critical analysis of included studies

LPS is widely used as an experimental model to study neuroinflammation, as it simulates the inflammatory response through the activation of glial cells, leading to the release of pro-inflammatory cytokines and triggering a neuroinflammatory cascade. The experimental models used in the studies included in this review applied LPS primarily via the intraperitoneal route, in doses ranging from 0.250 to 2 mg/kg. This model allows for the investigation of the underlying mechanisms of neuroinflammation and the evaluation of potential therapeutic agents that may mitigate inflammatory effects. The use of LPS in *in vivo* studies provides a deeper understanding of the interactions between peripheral and central inflammation, contributing to the development of strategies for treating neurodegenerative diseases associated with neuroinflammation.

Among the models analyzed in the studies, the vast majority investigated major depressive disorder, conducting behavioral analyses to assess depressive-like behavior (Han et al., 2021; Sun et al., 2021b; Singh et al., 2022; Adeoluwa et al., 2023; Tang et al., 2023; Su et al., 2024). Two studies focused on evaluating anxiety (Lee et al., 2020a; Singh et al., 2022), while two other studies utilized the LPS model focusing on PD (Kang et al., 2020; Han et al., 2021), and one study modeled age-related macular degeneration (Zou et al., 2024).

LPS has proven to be an important tool in investigating various pathologies, as it induces a systemic inflammatory response that mimics several clinical conditions. In studies related to depression, LPS is used to induce an inflammatory state that correlates with the neurochemical dysfunction observed in the disorder, allowing for the investigation of the mechanisms linking inflammation and mood alterations, since it has been found that individuals with depression exhibited an increase in the concentration of over ten pro-inflammatory cytokines (He et al., 2020). Similarly, in PD, LPS administration can lead to the death of dopaminergic neurons and microglial activation, causing neurodegenerative processes (Jiang et al., 2017). In the case of anxiety, it is known that inflammation and oxidative stress are involved in the pathophysiology of the disorder (Sulakhiya et al., 2016), and LPS can alter the activity of neurobiological pathways related to stress, enabling the analysis of the effects of inflammation on anxious behavior (Sulakhiya et al., 2016). Thus, the use of LPS as an experimental model is essential for understanding the relationship between inflammation and various diseases, contributing to the development of new treatments.

However, the number of studies investigating the anti-inflammatory effects of QUE or its derivatives in response to LPS is still somewhat limited. The small number of studies included in this review is due to the application of these inclusion criteria, specifically focusing on the effects of QUE and its derivatives in animals exposed to LPS. This approach was adopted to ensure data homogeneity and provide a more accurate analysis of the anti-inflammatory and neuroprotective effects of QUE in an induced inflammatory context.

Some methodological limitations were found in the included studies. The lack of standardization and methodological details can be attributed, at least in part, to the absence of consistent application of rigorous guidelines, such as those recommended by the ARRIVE checklist (Animal Research: Reporting of *In Vivo* Experiments). ARRIVE provides essential guidance for the planning, execution, and reporting of animal model studies, ensuring greater reproducibility and transparency. If the studies had followed these guidelines, there would have been more uniformity in experimental protocols, such as group design, dosage, routes of administration, treatment duration, and analysis parameters. This would reduce methodological variability across studies, facilitating direct comparison of results and generating more robust and reliable conclusions.

Among the studies, there was no standardized protocol regarding the duration of model induction or QUE treatment, which makes it difficult to establish the optimal dose and treatment duration. QUE demonstrated beneficial effects at doses ranging from 10 to 100 mg/kg over treatment periods ranging from 7 to 21 days, being administered orally (in most studies) or via intraperitoneal injection. Therefore, the variation in time intervals, doses, and administration routes complicates the identification of the ideal treatment conditions with the molecule. However, the data highlighted by the meta-analysis confirm the therapeutic validity and reliability of QUE treatment, regardless of the time and dose, in reducing neuroinflammation.

Despite the large number of preclinical evidence, there are currently no clinical trials investigating the potential benefits of QUE in neuroinflammation; only a few clinical studies involving QUE have been conducted to treat inflammatory conditions in general. A randomized clinical trial aimed at determining the safety of QUE supplementation in patients with chronic obstructive pulmonary disease, a condition characterized by chronic inflammation, administered QUE at doses of up to 2000 mg/day. The patients did not experience any serious adverse events related to QUE, as observed through complete blood counts and comprehensive metabolic panel assessments, indicating that QUE was safe and well-tolerated (Han et al., 2020). However, more large-scale studies are needed to confirm its efficacy and safety for long-term use.

Another ongoing clinical study (NCT05983224) aims to investigate the impact of QUE supplementation on factors such as glycemic status, lipid profile, oxidative stress, inflammation, and other hormonal factors in women suffering from endometriosis. Among the inflammatory factors to be investigated are the pro-inflammatory cytokines TNF-α and IL-6, measured using the enzyme-linked immunosorbent assay. The intervention group will receive two 500 mg QUE tablets daily, after breakfast and lunch, for twelve weeks. This clinical study has the potential to provide valuable insights into the role of QUE in biomarkers associated with inflammation and oxidative stress in humans.

## Conclusion and Perspectives

**Figure 7:**
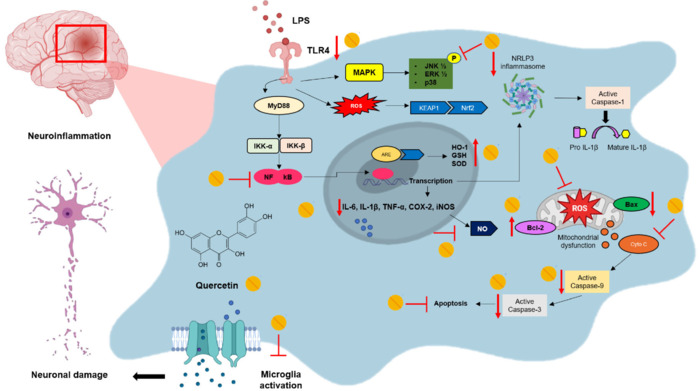
Mechanism of action by which QUE exerts anti-inflammatory, antioxidant, and anti-apoptotic effects in LPS-induced neuroinflammation. Neuroinflammation is triggered by the recognition of LPS on membrane receptors of microglial cells, such as TLR4 (Toll-like receptor 4), leading to the activation of multiple signaling pathways. The binding of LPS to the TLR4 receptor triggers a signaling cascade that activates adapter proteins like MyD88, resulting in the activation of IKK-α and IKK-β kinases. Once phosphorylated, these kinases are degraded by proteasomes, releasing NF-κB, which translocates to the nucleus and promotes the transcription of pro-inflammatory mediators (IL-6, IL-1β, TNF-α, COX-2, and iNOS). QUE, however, suppresses the translocation of NF-κB to the nucleus, reducing the production of inflammatory cytokines and decreasing TLR4 expression. Additionally, the MAPK signaling pathway (p38, JNK, and ERK) is stimulated by LPS, which further increases the production of pro-inflammatory mediators, inflammatory enzymes (COX-2 and iNOS), and reactive oxygen species (ROS). QUE inhibits the activation of this pathway and also prevents the activation of the NLRP3 inflammasome, thus inhibiting IL-1β maturation and reducing the inflammatory response. LPS also promotes ROS production, which activates the Keap1 protein, leading to the translocation of Nrf2 to the nucleus. QUE modulates this pathway by stimulating Nrf2 release, which, once in the nucleus, binds to antioxidant response elements (ARE) in the DNA, enhancing the expression of antioxidant genes such as HO-1, SOD, and GSH, which protect cells against oxidative stress. QUE reduces mitochondrial dysfunction and inhibits cytochrome C (Cyto C) release, thereby preventing the ROS-mediated apoptotic process. It decreases pro-apoptotic proteins such as Bax, while increasing the expression of anti-apoptotic proteins such as Bcl-2. Furthermore, QUE reduces the activation of caspases (such as caspase-3 and caspase-9), preventing excessive apoptosis induced by neuroinflammation. ‒‒|: inhibited/prevented; ↑: increased; ↓: decreased; Bax: BCL2 associated X apoptosis regulator; Bcl-2: BCL2 apoptosis regulator; COX-2: cyclooxygenase-2; Cyto.c: cytochrome c; ERK1/2: serine/threonine kinases 1 and 2; GSH: reduced glutathione; HO-1: heme-oxygenase 1; IL: interleukins; JNK: c-Jun N-terminal kinase; LPS: lipopolysaccharide; MAPK: mitogen-activated protein kinase; MyD88: myeloid differentiation primary response 88; NLRP3: NOD-like receptor family pyrin domain containing 3; NO: nitric oxide; Nrf2: nuclear factor erythroid 2-related factor 2; p38: mitogen-activated protein kinase; QUE: quercetin; ROS: reactive oxygen species; SOD: superoxide dismutase; TNF-α: tumor necrosis factor alpha; iNOS: nitric oxide inducible.

This systematic review gathered relevant information about the therapeutic potential of QUE and its derivatives in the treatment of LPS-induced neuroinflammation. It was observed that QUE and its derivatives demonstrated the ability to modulate the inflammatory response in various preclinical models. Additionally, information was collected on the doses used and the different exposure times, thereby providing crucial data for future studies. The beneficial effects of QUE and its derivatives primarily occur due to their ability to modulate key pathways involved in neuroinflammation, such as NF-κB, which reduces the production of inflammatory mediators; MAPK, including ERK, JNK, and p38; the Nrf2 pathway; and the modulation of microglial activity, along with mechanisms based on their antioxidant and anti-apoptotic responses (**Figure 7**).

As mentioned earlier, some limitations, such as extensive metabolism and low bioavailability, may hinder the therapeutic use of QUE. Consequently, researchers in drug development have made efforts to address these challenges, including through structural modifications or the use of nanoparticles (Arbo et al., 2020; Rifaai et al., 2020; Sanad et al., 2023).

However, despite the promising results in animal models, future directions should focus on well-designed clinical studies to assess the safety, bioavailability, and efficacy of QUE in humans. Additionally, standardization of methods and dosages in studies is crucial to ensure the consistency of findings and optimize their application in clinical settings. Future studies may also explore the development of new QUE derivatives or combinations with other anti-inflammatory agents to maximize their therapeutic potential. We also recommend that more attention should be paid to investigating the optimal concentrations, the exact duration of treatment, and the efficiency of interventions in clinical trials using QUE and its derivatives in the neuroinflammatory context.

## Additional files:

***Additional file 1:*** *Open peer review report 1.*

OPEN PEER REVIEW REPORT 1

***Additional Table 1:*** *Extraction of data from studies.*

Additional Table 1 Extraction of data from studies

## Data Availability

*Not applicable*.
