# Supplementary material for: Effects of quercetin and its derivatives in in vivo models of neuroinflammation: A systematic review and meta-analysis
Source: Neural Regen Res. 2025 Mar 25;21(5):1783–92. doi: 10.4103/NRR.NRR-D-24-01175 (PMC12694622; doi:10.4103/NRR.NRR-D-24-01175)
Supplement: Supplementary file 1 [file NRR-21-1783_Suppl2.pdf]

Additional Table 1 Extraction of data from studies

First author (year)	Species (sex/n)	Age/weight	Quercetin/ Derivatives (dose/route of administration)	Quercetin/ derivatives treatment (type/ exposure time/frequency)	LPS (dose/time/route of administration)	Methods/evaluated parameters	Anti-neuroinflammatory activities (primary outcomes)	Secondary outcomes
**Adeoluwa et al., 2023**	Male rats (*n* = 6-7/group)	17–18 weeks<br>-	QUE<br>50 mg/kg<br>V.O	Pretreament<br>7 days	0.85 mg/kg<br>1x<br>I.P.<br>1 day	-FST<br>-SST<br>-OFT<br>**ELISA** (TNF-α, IL-17, IL-6)<br>**IHC** (iNOS, NF-κB, AIF)<br>Inflammasome expression.	↓ IL-17, IL-6, TNF-α (hippocampus)<br>↓TNF- α (cortex)<br>↓ AIF-1, NLRP3, iNOS, NF-kB (cortex and hippocampus).	↑ Mobility time in FST<br>↑ Grooming time in SPT<br>→ Spontaneous motor activity in OFT
**Hussein et al., 2024**	Male BALB/c mice (*n* = 6-10 group)	-<br>26.5–32.0 g	QUE<br>50 mg/kg<br>V.O<br>QUE NANO: 50 mg/kg<br>V.O	Post-treatment<br>7 days	0.250 mg/kg/d<br>I.P.<br>7 days	-Histological analysis of the brain<br>-GSH<br>-MDA<br>-CAT<br>**-ELISA:** GABA, Aβ42, β-secretase and iNOS<br>-AChE activity;<br>**-qRT-PCR:** Nrf2, HO-1	↓iNOS levels	↓ vascular degeneration<br>↑ GSH level and CAT activity.<br>↓ MDA<br>↑ HO-1<br>↑ Nrf2<br>↓ Aβ-42 and β-secretase;<br>↓ AChE activity<br>↑ GABA levels
**Kang et al., 2020**	Male C57/BL6 mice (*n* = 5/ group)	8 weeks 19–23 g	QUE<br>1 or 10 mg/kg/day<br>V.O	Pretreatment<br>3 days	10 mg/kg<br>I.P.<br>1 day	**-IHC:** GFAP- or Iba-1-positive cells (hippocampus or substantia nigra)<br>TH	↓ activation of astrocytes (10 mg/kg) or microglia (1 mg/kg) SN and hippocampus	↑ TH positive cells and fibers
**Khan et al., 2018**	Male C57BL/6N mice (*n* = 15/ group)	8 weeks, 25–30 g	QUE<br>30 mg/kg/day<br>I.P	Pretreatment and co-treatment<br>2 weeks	0.25 mg/kg/day,<br>I.P.<br>7 days	-MWM<br>-Y-maze task<br>**-WB:** Iba-1, GFAP, pNF-κB, TLR-4, PSD, Synap, TNF-α, NOS-2, COX-2; ADP-ribose; PARP-1, Cyto.c<br>**-IHF:** GFAP, p-NF-kB, IL-1β, caspase-3, SNAP23	↓ GFAP, Iba-1<br>↓ pNF-κB (cortex and hippocampus)<br>↓ TNF-α, COX-2, NOS-2 and IL-1β<br>↓ TLR-4 (cortex and hippocampus)	↑ PSD95 and Synap (cortex and hippocampus)<br>↑ SNAP-23 (cortex and hippocampus)<br>↓ Bax, Cyto.c, PARP-1 and caspase-3 (cortex and hippocampus)<br>↑ Bcl-2 (cortex and hippocampus)

								↑ Memory performance (MWM task and a Y-maze task)<br>↑ Survival of neuronal cells in the cortex<br>and hippocampus
**Lee et al., 2020**	Male Sprague-Dawley rats (*n* = 3/group)	6 weeks<br>210–230 g	QUE<br>10, 50 and 100 mg/kg<br>I.P.	Post-treatment<br>21 days	2 μL/1 min for 5 min<br>I.C.V.	-OFT<br>-Elevated Plus-Maze Test;<br>-Contextual Fear Conditioning and Extinction;<br>**-ELISA:** IL-6, IL-1β, TNF-α, COX-2, and NF-κB<br>**-PCR:** BDNF, TLR4, and iNOS	↓ IL-6, IL-1β, COX-2, NF-kB<br>→TNF-α<br>↓ iNOS<br>→TLR-4	QUE 100 mg/kg<br>↑ Body weight through days 14 and 21<br>↓ Anxiogenic parameters<br>↓ Anxiety index<br>↑ The number of crossings in the central zone<br>↓ Grooming behavior (OFT)<br>→ Freezing time on days 7 and 14<br>↓ Freezing time on day 22.<br>↑ BDNF expression
**Su et al., 2024**	Male<br>ICR mice (*n* = 10)	-<br>20 ± 2 g	QUE<br>25 and 50 mg/kg/day<br>I.G.	Pretreatment<br>14 days	1 mg/kg<br>1 day<br>I.P	-OFT<br>-SPT<br>-TST<br>-The mast cell activation<br>**-IHC**: β-tryptase<br>**-WB:** β-tryptase protein levels		↑ The sucrose preference in SPT<br>↓ The immobility time in TST<br>→ The total distance of mice in OFT<br>↑ The time of staying in central area in OFT (QUE 50 mg/kg)<br>↓ number of mast cells (hippocampus)<br>↓ β-tryptase positive cells in the hippocampus

**Singh et al., 2022**	Male and Female Zebrafish (*n* = 12/ group)	3 months<br>470–530 mg	QUE<br>50 and 100 mg/kg/d<br>I.P	Post-treatment<br>7 days	1 mg/kg<br>1 day<br>I.P	-Novel tank diving test;<br>-Light–dark chamber test;<br>-LPO, GSH, nitrite, AChEs activity;<br>**-ELISA**: TNF- α and IL- 1β<br>Histopathological analysis	↓ TNF-α and IL-1β<br>↓ Nitrite	↑ Time TSTZ (Novel tank diving)<br>↓Time TSBZ (Novel tank diving)<br>↑ The number of entries in the top zone<br>↑ Time TSLZ (light–dark chamber)<br>↓ Time TSDC (light–dark chamber)<br>↑ The number of entries in the light zone<br>↓ The loss of neuronal cell<br>↓ LPO, AChEs<br>↑ GSH
**Han et al., 2021**	Male C57BL/6J mice (*n* = 4–9/group)	3 months<br>-	QUE<br>30 and 60 mg/kg/d<br>I.P.	Pretreatment and co-treatment<br>5 days (depression model)<br>7 days (DP model)	**DP model:**<br>0.5 μg/1 μL (0.2 μL/min)<br>1 day<br>I.C.V;<br>**Depression model:**<br>1 mg/kg/d<br>5 days<br>I.P	-FST<br>-TST<br>-The rotarod test;<br>**-WB**: NLRP3, caspase-1, pro-IL-1β, IL-1β and PINK1 (hippocampal and mesencephalic)<br>-IF: Iba-1<br>-IHC: TH in SNpc sections	**Depression model:**<br>↓ NLRP3, IL-1β, and IBA-1$^+$ cells;<br>**DP model:** ↓ NLRP3, IL-1β and IBA-1$^+$ cells,	**Depression model:**<br>↓ Immobility time in FST and TST<br>↓ Caspase-1<br>**DP model:**<br>↓ Caspase-1<br>↑ TH, number TH$^+$ cells and latency to fall (QUE 60 mg/kg)
**Zou et al., 2024**	Male and female C57BL/6 J mice (*n* = 4/ group)	8-10 weeks<br>-	QUE<br>30 mg/kg/day<br>I.P	Pretreatment and co-treatment<br>4 days	2 mg/kg<br>1 day<br>I.P	**-IF**: Iba-1, IB4, iNOS, ZO-1, CD11b, IgG<br>**-RT- qPCR**: CD206, CD16, IL-10, iNOS, IL-1β, IL-6, TNF-α, Arg1, β-actin);<br>**-WB:** Occludin ERK Ab, pERK, β-actin, GAPDH, Evans Blue Leakage Assay	↓ The number of IBA1-positive cells in the OPL,<br>→ The number of IBA1-positive cells in IPL<br>↓ iNOS, IL-1β, IL-6, and TNF-α;<br>↑ IL-10<br>↓The mRNA expression levels of M1 markers, CD16<br>↑ The expression of M2 markers, CD206, Arg1	↓ Inflamed retinal vascular leakage<br>↑ Occludin expression

**Tang et al., 2023**	Male Sprague-Dawley rats (n = 30-31/ group)	5 weeks	**AGIQ** 0,5% (p/p) of the diet	Pretreatment and co-treatment 38 days	1 mg/kg/day 2 days I.P	-OFT<br>-Contextual fear conditioning test,<br>**-ELISA:** IL-1β, IL-6, TNF-α, MDA<br>**-IHC:** GFAP, SOX2, TBR2, DCX, TUBB3, NeuN, PVALB, RELN, CALB2, GAD67, SST, PCNA, ARC, COX-2, Iba1, CD68, CD163; apoptotic cells (TUNEL).	Hippocampus and cerebral cortex<br>↓ TNF-α<br>→ IL-6. IL-1β,<br>→ Iba-1, GFAP<br>↑ COX-2	→ Total distance,<br>→ Contextual fear conditioning test<br>↑ MDA<br>→ CD68, CD163<br>→ SOX2 $^{+}$, TBR2^{+}, TUBB3^{+}, NeuN^{+},<br>↑ PCNA^{+}, FOS^{+}<br>→ ARC^{+}, DCX<br>↑ CALB2^{+}<br>→ PVALB^{+}, RELN^{+}, GAD67^{+} SST^{+}<br>↓ Sod1<br>→ Granule cell lineage marker genes (except ↓ Dcx)<br>→ GABAergic interneuron marker genes<br>→ Neurotrophic factor-related genes<br>→ Synaptic plasticity-related IEGs<br>→ Glutamate receptor and transporter genes<br>→ Cholinergic receptor genes
**Sun et al., 2021b**	Male ICR mice (n = 5−12/group)	6−8 weeks 22 −24 g	Quercitrin (Qc) 10 mg/kg I.P	Post-treatment 1 day	0.5 mg/kg/day 5 days I.P	-OFT<br>-TST<br>-FST<br>-SPT<br>**-WB**: pERK, ERK, pJNK, JNK, pP38, P38, pPI3K, PI3K, pAKT, AKT, pNF-kB, NF-kB<br>**-ELISA**: IL-1β, IL-10, TNF-α	**Qc (10 mg/kg)**<br>↓ IL-1β, IL-10, TNF-α hippocampal<br>↓ pPI3K/PI3K, pAKT/AKT, pNF-κB/NF-κB<br>↓ pERK/ERK, pP38/P38, pJNK/JNK	**Qc (10 mg/kg)**<br>↓ Immobility time in the TST and FST,<br>↑ Sucrose preference significantly<br>→ The time spent in center and total area (OFT)

-: Does not contain the information; **—|:** inhibited/prevented; **↑:** increased; **↓**: decreased; **→**: not affected; Aβ: amyloid-beta 42; AChE: acetylcholinesterases; ADP-ribose: adenosine diphosphate ribose; AGIQ: α-glycosyl isoquercitrin; AIF: apoptosis-inducing factor; Akt 1: AKT serine/threonine kinase 1; AMPK: adenosine monophosphateactivated protein kinase; AP-1 transcription factor subunit; ARC:

cytoskeleton-associated protein; Arg: arginine; Bax: BCL2 associated X apoptosis regulator; ; Bcl-2: BCL2 apoptosis regulator; BDNF: brain-derived neurotrophic factor; CALB1: calbindin-D-28K; CALB2: calbindin-D-29K; CAT: catalase; CD68 or CD163: cluster of differentiation; CNPase: 2',3'-cyclic nucleotide 3'-phosphodiesterase; COX-2: cyclooxygenase-2; Cyto.c: Cytochrome c; DCX: doublecortin; DA neurons: dopaminergic neurons; DMSO: dimethyl sulfoxide; ERK1/2: serine/threonine kinases 1 and 2; FOS: Fos proto-oncogene ; FST: forced swimming test; GAD67: glutamic acid decarboxylase 67; GABA: gamma-aminobutyric acid; GFAP: glial fibrillary acidic protein; GSH: reduced glutathione; GSSG: glutathione disulfide; HO-1: heme-oxygenase 1; I.C.V: intracerebroventricularly; I.G: intragastric; Iba-1: ionized calcium-binding adapter molecule 1; IF: immunofluorescence; IHC: immunohistochemistry; IL: interleukins; iNOS: nitric oxide inducible synthase; I.P: intraperitoneal; IPL: internal plexiform layer e externa; JNK: c-Jun N-terminal kinase; LPO: estimation of lipid peroxidation; LPS: lipopolysaccharide; MAPK: mitogen-activated protein kinase; MWM: Morris water maze; NeuN: neuronal nuclei; NG2: sulfate proteoglycan; NLRP3: NOD-like receptor family pyrin domain containing 3; Nrf2: nuclear factor erythroid 2-related factor 2; NO: nitric oxide; NOS-2: nitric oxide synthase 2; OFT: open field test; OLIG2: oligodendrocyte lineage transcription factor 2; OPL: outer plexiform layer; P38: mitogen-activated protein kinase; p-AKT1: serine/threonine kinase phosphorylated; PARP: poly-ADP-ribosylation; PCNA: proliferating cell nuclear antigen; PCR: polymerase chain reaction; PD: Parkinson's disease; PINK1: PTEN-induced kinase 1; PSD: postsynaptic density; PVALB: parvalbumin; QC: quercitrin; QUE: quercetin; RELN: reelin; ROS: reactive oxygen species; SOX2: SRY-box transcription factor 2; SPT: sucrose preference test; SST: sucrose splash test; STAT3: signal transducer and activator of transcription 3; Synap: synapse; TB: toluidine blue; TBR2: Tbox brain protein 2; TH: tyrosine hydroxylase; TLR4: Toll-like receptor 4; TNF-α: tumor necrosis factor alpha; TSBZ: time spent in the bottom zone; TST: tail suspension test; TSTZ: time spent in the top zone; TUBB3: tubulin, beta 3 class III; V.O: oral route; WB: western blot assay.
