# Supplementary material for: Effects of quercetin and its derivatives in in vivo models of neuroinflammation: A systematic review and meta-analysis
Source: Neural Regen Res. 2025 Mar 25;21(5):1783–92. doi: 10.4103/NRR.NRR-D-24-01175 (PMC12694622; doi:10.4103/NRR.NRR-D-24-01175)
Supplement: Supplementary file 2 [file NRR-21-1783_Suppl1.pdf]

OPEN PEER REVIEW REPORT 1

Name of journal: Neural Regeneration Research
Manuscript NO: NRR-D-24-01175
Title: Effects of quercetin and its derivatives in in vivo models of neuroinflammation: a systematic review and meta-analysis
Reviewer's Name: Stefania Ceruti
Reviewer's country: Italy

COMMENTS TO AUTHORS

Overall evaluation on article quality:
With this manuscript authors provide a systematic review and metanalysis on in vivo preclinical studies showing the effects of the flavonoid Quercetin in neuroinflammatory conditions induced by LPS administration.

Overall, the topic of this review and metanalysis is very interesting, based on the clear demonstration of a role for neuroinflammation in many brain pathologies and on the growing interest on the use of naturally-occurring compounds to blunt this condition.
Although the number of included studies is limited, mostly due to an overall low quality of other manuscript and to the strict inclusion criteria correctly set up by the authors, results of the metanalysis confirm the anti-inflammatory action of Quercetin and its ability to revert microglia activation under inflammatory conditions. Additionally, this analysis highlights several limitations in pre-clinical studies (e.g., the lack of adherence to the ARRIVE guidelines) which must be taken into serious considerations to improve the quality and significance of animal pre-clinical research.

I have some comments and suggestions for the authors to further improve the quality of their manuscript, as listed below.

1. Page 5, lines 7-8: the correct IUPAC name of quercetin is 2-(3,4-dihydroxyphenyl)-3,5,7-trihydroxychromen-4-one. Please, modify.
2. Abbreviations should be explained the first time they are used and from there on must be utilized throughout the manuscript.
3. Page 7, line 14: since Quercetin has many different common and chemical names, why were just two of them included in the search criteria? Please, explain.
4. Results and Figure 2: authors have found more than 13,000 "grey" papers in Google Scholar but have decided to analyze "only the first 100". What does this mean? How were papers rated? Were those papers the most recent 100 manuscripts? Please, clarify.
5. Page 18, line 2: I guess that a reference is missing when (REF) is written. Conversely, in page 19, lines 34-35 a reference is incorrectly quoted as "Priyanga K and K, 2017".

Timeliness evaluation on article:
Very good evaluation to existing literature. Please, refer to point #4 above.

Scope evaluation on article:
Please, refer to my first comment above

Direction evaluation on article:
Please, refer to my first comment above
